# Preliminary Evaluation of Sedentary Lifestyle in Italian Children after Solid Transplant: What Role Could Physical Activity Play in Health? It Is Time to Move

**DOI:** 10.3390/ijerph20020990

**Published:** 2023-01-05

**Authors:** Eliana Tranchita, Giulia Cafiero, Ugo Giordano, Isabella Guzzo, Raffaella Labbadia, Stefano Palermi, Claudia Cerulli, Manila Candusso, Marco Spada, Lucilla Ravà, Federica Gentili, Fabrizio Drago, Attilio Turchetta

**Affiliations:** 1 Department of Cardiac Surgery, Cardiology and Heart Lung Transplant, Division of Sports Medicine, Bambino Gesù Children’s Hospital IRCCS, 00165 Rome, Italy; 2 Kidney Transplant Follow-Up Unit, Division of Nephrology, Department of Pediatrics, Bambino Gesù Children’s Hospital IRCCS, 00165 Rome, Italy; 3 Public Health Department, University of Naples Federico II, 80131 Naples, Italy; 4 Unit of Physical Exercise and Sport Sciences, Department of Movement, Human and Health Sciences, University of Rome Foro Italico, 00135 Rome, Italy; 5 Hepatology, Gastroenterology, Nutrition and Liver transplantation Unit, Bambino Gesù Children’s Hospital IRCCS, 00165 Rome, Italy; 6 Division of Abdominal Transplantation and Hepato-Bilio-Pancreatic Surgery, Bambino Gesù Children’s Hospital IRCCS, 00165 Rome, Italy; 7 Clinical Epidemiology Unit, Bambino Gesù Children’s Hospital IRCCS, 00165, Rome, Italy; 8 Paediatric Cardiology and Cardiac Arrhythmias Unit, Department of Cardiac Surgery, Cardiology and Heart Lung Transplant, Bambino Gesù Children’s Hospital IRCCS, 00165 Rome, Italy

**Keywords:** transplant, children, physical exercise, health

## Abstract

Background: Advances in the medical–surgical field have significantly increased survival after solid organ transplantation in the pediatric population. However, these patients are predisposed to the development of long-term complications (e.g., cardiovascular disease). The therapeutic role of physical activity (PA) to counteract these complications is well known. The purpose of the study was to investigate the level of PA in a pediatric population after solid organ transplantation. Methods: In the first 4 weeks at the beginning of the school year, the Physical Activity Questionnaire for Older Children and Adolescents was administered to young patients who had previously undergone solid transplants at our institute. Results: Questionnaires of 49 patients (57.1% female, mean age 13.2 ± 3.5 years) were analyzed and 32.7% of subjects did not perform any exercise during school physical education classes. Only 24% practiced a moderate quantity of exercise in the previous week (2–3 times/week) and 72% engaged in sedentary behaviors during weekends. Conclusions: Preliminary data confirmed that young recipients are still far from meeting the minimum indications of the World Health Organization on PA and sedentary behavior. It will be necessary to increase their involvement in PA programs in order not only to increase their life expectancy but also to improve their quality of life.

## 1. Introduction

Solid organ transplantation in the pediatric population is one of the most important achievements of modern medicine and it allows many children the opportunity to have a normal life. Liver transplantation (LTx) is a life-saving treatment for children with end-stage liver disease or metabolic disease with hepatic localization [1]. According to the 2018 report [2] of the European Liver Transplant Registry (ELTR), 146.762 liver transplants were performed in Europe until 2016, and approximately 2% of them were performed in children [3]. Kidney transplant (KTx) is another type of intervention that involves the pediatric population. Recent data from the European Renal Association Registry (ERA) show that a total of 24.013 kidney transplantations were carried out in 2019 in 34 European–Mediterranean countries, 3% of them involving young patients [4]. KTx is the optimal treatment for end-stage kidney failure due to several causes both in adults and in younger patients [5,6]. It is important to understand that as the survival rate increases, at the same time, it increases the risk of developing further diseases in these patients, often due to the medications used [7]. In particular, LTx can lead to kidney injury, diabetes, infectious diseases, deficits or delays in childhood development. When used, high doses of corticosteroids predispose patients to growth and bone mineralization deficits, causing osteoporosis and a lower height in children; moreover, the use of these medications increases the risk of hypertension and diabetes in children [7,8]. Using calcineurin inhibitors (cyclosporine or tacrolimus) can cause chronic kidney failure, infections and gastrointestinal diseases [8,9]. Lastly, but no less important, immunosuppressive therapies increase remarkably the risk of cancer onset, such as cancer of the skin/soft tissue, but also lymphoma, leukemia and diseases of other organs [7,10,11]. Studies on solid organ transplantation highlighted that these children have an increased cardiovascular risk. They can develop hypertension, cardiac left ventricular hypertrophy, obesity, dyslipidemia and diabetes, too [12,13]. At the same time, children with KTx use immunosuppressive drugs and corticosteroids that, similarly to what happens in LTx, can cause an increased risk of infection, osteoporosis and secondary malignancies [6,14].

Physical activity (PA) may represent a valid tool to prevent cardiovascular risk factor onset, osteoporosis [15], kidney failure and cancer [13]. PA also represents a therapeutic tool that can restore both physical, functional and psychological capacities, and it was demonstrated to be safe, feasible and effective [16] to prevent the decline in the quality of life in children who underwent transplantation. In Italy, in 2008, a specific program named “Trapianto…e adesso sport” (i.e., “transplant… and now it is time for exercise”) was introduced, aiming at highlighting the importance of PA after surgery among transplant recipients [17]. However, this study considered only patients who received transplants in adulthood and was conducted on a population aged over 18 years old, thus excluding children and adolescents [18,19]. At this time, there are no similar projects aimed at the pediatric population in Italy. Recent studies [20] demonstrated that children who received LTx reported more sedentary hours and more daily hours on the computer than healthy controls. More recently, some studies have been conducted to demonstrate the beneficial effects of physical exercise on KTx recipients’ health. Practicing regular physical activity, even with low exertion, can improve cardiorespiratory fitness [21] in these children, and consequently their quality of life improves. It is therefore necessary to focus on improving post-transplant management by adding physical activity as a non-pharmacologic tool, in order to guarantee better health and quality of life, especially in younger transplant recipients.

The aim of the present study was to assess the levels of PA in children who received liver or kidney transplants. This represents a preliminary stage in understanding how many of them should be urged to start or restart the practice of physical exercise after a solid organ transplant, with the ultimate goal of improving their health and their quality of life, and increasing their survival.

## 2. Materials and Methods

### 2.1. Study Design

A cross-sectional observational study was carried out over a 6-week period. For the scope of the study, a questionnaire was administered to evaluate how much PA young transplant patients practiced. In the first four weeks of the beginning of the school year, patients were asked to complete the questionnaire during post-transplant follow-up visits and they voluntarily decided to answer the questions.

The study conformed to the ethical principles of Good Clinical Practice and the Helsinki Declaration, it was approved by the Ethics Committee of Bambino Gesù Children’s Hospital (protocol code 2070_OPBG_2020), and it followed the current Italian regulations. Moreover, all subjects and their parents were verbally informed about the aim and the procedures of the study. All participants were assured about the anonymity of data and that the data would be processed for scientific purposes and in an aggregate manner only.

### 2.2. Participants

From the total population of children who underwent LTx and KTx between 2010 and 2021 at Bambino Gesù Children’s Hospital in Rome (n. 545 patients), we selected the study population based on the following inclusion criteria:

- Age between 8 and 18 years old;
- At least 2 years after the intervention (range 2–5 or more years);
- Stable clinical condition.
- Exclusion criteria were
- Patients unable to complete the questionnaire due to age and/or psycho-physical limitations;
- Patients with a history of congenital heart disease, even if corrected and cured.

Children with congenital heart disease were not enrolled to avoid the confounding effects of other pathologies on exercise practice.

### 2.3. Questionnaire

All patients filled out the questionnaire “Physical Activity Questionnaire for Older Children (PAQ-C) and Adolescents” (PAQ-A) [22] to evaluate the amount of physical activity that they practiced spontaneously. This questionnaire was recently validated for the Italian language [23]. It is a seven-day-recall self-administered questionnaire to evaluate the amount of moderate to vigorous physical activity practiced by school-aged children. In particular, PAQ-C and PAQ-A consisted of 10 items and they were scored by means of 9 items, rated between 1 (low physical activity) and 5 (high physical activity); see Table 1. 

### 2.4. Statistical Analysis

Statistical analysis was performed using STATA 17.1 software. Categorical data were reported as counts and proportions; continuous data were represented as mean and standard deviation or median and interquartile range. Comparisons of categorical variables between groups were performed through the Chi-square test or Fisher exact test, while, for continuous variables, the Student *t*-test for independent data was used. The Student *t*-test for dependent data was used in order to compare continuous variables at time 0 and time 180. Results were considered statistically significant for *p*-values < 0.05.

## 3. Results

**Figure 1:**
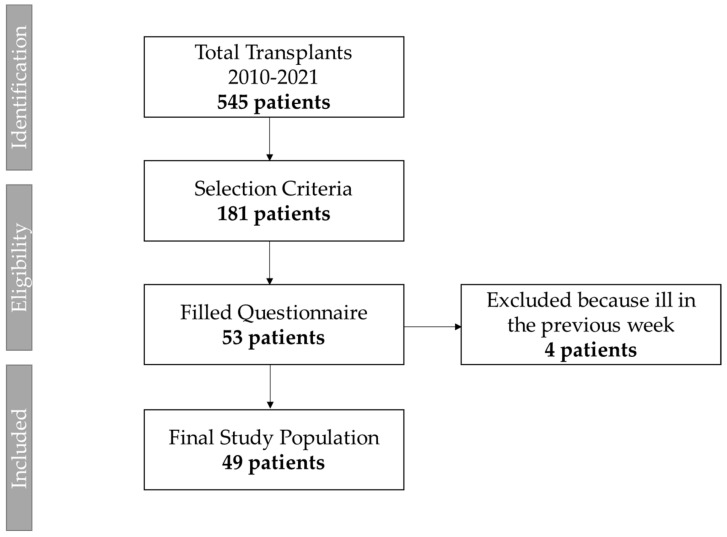
Study flow-chart.

From the total of 545 subjects who underwent transplants at our hospital, a total of 53 subjects voluntarily filled out the questionnaire. Four of them were excluded from the analysis because they were sick in the previous week, so the data collected through the questionnaire would not have been representative of their daily life. Therefore, data from 49 patients were used for the purpose of the present study (Figure 1).

Characteristics of the sample are shown in Table 2. The mean age of the children was 13.2 ± 3.5 years old (range 8–18) and most of them were female (57.1%) and of a normal weight (mean body mass index was 20 ± 3.6 kg/m^2^). Approximately 65% of children received liver transplantation, 30% underwent kidney transplantation and less than 5% received both.

**Figure 2:**
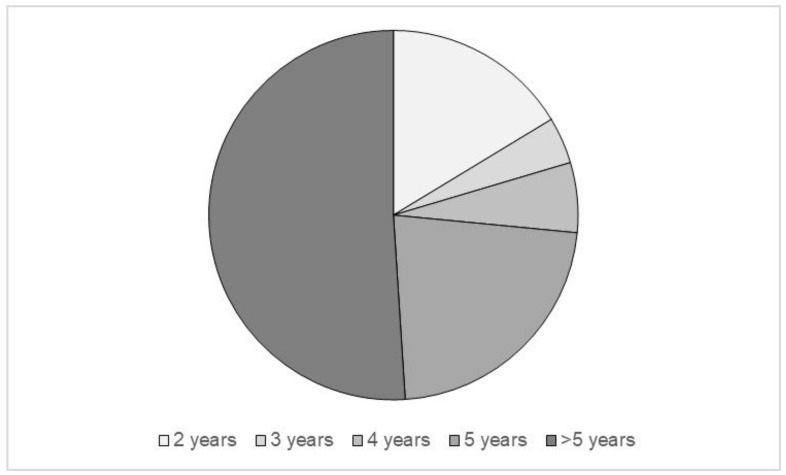
Time elapsed since transplantation.

Almost half of the cases received the transplant more than 5 years ago (51%), as shown in Figure 2.

The most frequent activities that the children engaged in in their spare time were walking or running, at least 2 or 3 times/week, followed by jumping.

Considering physical exercise, practiced during very active physical education (PE) classes, the most frequent answers were “I don’t do PE” (33%) and “Quite often” (24%). Conversely, most of the time, at recess, children sat down or stood around and walked around (37% each). No respondent stated that they ran and played hard most of the time. Similarly, concerning activities during lunch time, the most frequently reported answers were sitting down (talking, reading, doing schoolwork) and standing around or walking around (53% and 27%, respectively). Again, no respondent stated that they ran and played hard most of the time. In the afternoon, 45% of children practiced very active sports, dance or games 2 or 3 times a week, whereas, in the evening, this percentage dropped to 29% and, in most of the cases (45%), no activity was reported. Similarly, during the weekend, very active sports, dance or games were seldom practiced (31% 1 time, 41% none). The general self-perception of the degree of sedentary lifestyle expressed by the children was coherent with the previous answers, with little, 1–2 times or 2–3 times a week as the most frequently reported answers (24%, 27%, 24%, respectively). Details of answers are reported in Table 3.

The average score recorded in our population was 2.41 ± 0.12 (median 2.30, IQR 1.80–2.70). Significant statistical differences in average scores were not observed between groups when we compared patients based on gender, age, type of transplant, time elapsed since transplant or body mass index; see Table 4.

## 4. Discussion

The objective of this cross-sectional observational study was to investigate how many children who receive a liver or kidney transplant are physically active, through the use of validated questionnaires. The main results of the study suggested that most of the children who received LTx or KTx tended to have sedentary behavior. The present study was conducted on children during the first 4 weeks of the school year, without any form of restriction due to the COVID-19 pandemic. All school and recreational activities were fully accessible and it was possible to practice outdoor activities due to the mild climate. 

The average score recorded in our population (mean 2.41 ± 0.12, median 2.30 IQR 1.80–2.70) was similar to the scores previously reported by Lui [24] (median 2.2, IQR 1.7–2.9), Hamiwka [25] (mean 2.8 ± 0.8) and Patterson [26] (median 3.1, IQR 2.60–3.51), who examined physical activity experiences in children post-transplant using the same questionnaire. Comparing the average scores observed in our population with those of healthy subjects of the same age and gender (Voss et al.) [27], significant statistical differences were not observed. Nevertheless, 74% of the children observed in the present study showed a lower score based on that expected for their age and gender.

The main results of the study suggested that most of the children who received LTx or KTx practiced unstructured PA in their spare time, such as walking, running and jumping. Fifty-three percent of them showed a low level of exercise during physical education classes. Most of them showed an inactive attitude during recess (37%) or lunch (53%). Fortunately, they practiced moderate physical activity (2–3 times/week) in the afternoon after school-time (45%) but they did so less frequently in the evening (29%). The children preferred practicing sports activities in the central part of the week, while they were sedentary during the weekend, when 72% of them did not practice any type of activity or at most they exercised one time. Only 24% of children who received LTx or KTx practiced a moderate quantity of exercise in the previous week (2–3 times/week) and only 8% were engaged in very frequent activity (7 or more times last week) during their free time. A great number of them tended to display sedentary behavior on most days of the week, particularly during weekends. Therefore, these children were very far from meeting the amount of PA recommended by guidelines [28], often not met also by healthy subjects [29]. 

Studies in the literature [20] demonstrated that LTx children reported more sedentary hours than healthy controls. For example, 35% percent of LTx recipients spent 3 or more hours on the computer daily, compared to 22% among their non-transplant peers. Studies on children (7–20 years) with renal chronic disease [30] showed that only 10% of the male and 5% of the female participants met the recommendations [31] for pediatric populations of 15,000 and 12,000 steps per day, respectively. More recently, other studies have demonstrated the beneficial effects of PA on KTx recipients’ cardiorespiratory fitness and quality of life [21]. It was established in the adult population that structured exercise can ameliorate the metabolic profile [32] and body composition in KTx patients and lead to significant improvements in aerobic fitness, muscle strength and quality of life [33], with a significant decrease in body mass index. Thus, lifestyle modifications, including the intensification of physical exercise, are of great importance to improve the outcome after pediatric renal transplantation. It is therefore necessary to integrate post-transplant management with PA, with the aim of improving health and quality of life, in younger transplant recipients. The Italian project “Trapianto…e adesso sport” first highlighted, in our country, the importance of exercise in adult transplant recipients, and, at the same time, it was important to collect data about the conditions of transplant recipients, to study and measure the effects of sports and physical activity in this particular population. The results of this project confirm that physical activity, prescribed by specialist doctors and administered by specialized personnel (kinesiologist specialists in preventive and adapted motor activity), is able to improve both the biological parameters and the physical condition of the transplant recipient [17].

For the purpose of the study, we administered the PAQ-C and A questionnaires. These are self-reported questionnaires that were chosen because they are inexpensive and easy to administer and they gave us the opportunity to collect, anonymously, contextual details about physical activity that cannot be obtained through objective measures. Among several questionnaires for children, the PAQ-C and A seem to be promising [34]; they have been widely used in research and school settings in children aged 8–14 years [35], even if they should not be used to evaluate physical activity in the summer or holiday periods [22]. The PAQ-C and A assess activities related to leisure, common sports and physical education classes [34,35]. The PAQ-C and A demonstrated acceptable psychometric properties [34,35], with acceptable-to-good internal consistency, test–retest reliability and sensitivity to detect gender differences [35,36,37,38,39]. Moreover, these questionnaires demonstrated convergence with athletic competence, enjoyment perception, body mass index and cardiorespiratory and cardiovascular fitness [23,36,37,38,40]. Nevertheless, it is important to keep in mind that the PAQ-C and A questionnaires provide only self-report measures of how much physical activity these children practice. It is possible that what is reported is not entirely accurate, but that it corresponds to their perceptions of how much physical activity they practice. Probably, these data should be associated and/or compared with a more objective measurement of physical activity, which could be achieved through pedometers or other wearable devices that measure the amount of physical activity practiced, so as to most accurately quantify associations between physical activity and health [41]. Recent studies showed a moderate relationship between PAQ-C and wearable device (accelerometer) measurements and recommended that we concurrently administer both tools to obtain a more complete and realistic view of children’s PA, in terms of quality and quantity [42].

Admittedly, our study suffers from some limitations, such as the small sample size and the lack of a control group of healthy subjects. However, these preliminary results highlight that the problem of a sedentary lifestyle in post-transplant patients must be addressed as early as possible, especially with a view to preventing further complications in these patients. Furthermore, we did not investigate any existing comorbidities, although we excluded children with major cardiovascular complications.

On the other hand, one of the strengths of the present research was that, for the first time in Italy, a health assessment of children after receiving transplants was performed, including a lifestyle assessment. In particular, the amount of sport that these children practice has been quantified using a simple, immediate and repeatable tool, namely a questionnaire. This has enabled us to improve the quality of our clinical evaluations in post-transplant children, allowing us to obtain a more complete assessment of their health status. Attempting to determine which clinical factors may contribute to the low physical activity levels might also be helpful to reduce any factors that may hinder the practice of physical activity.

## 5. Conclusions

This preliminary work provides additional evidence that most children have relatively low levels of physical activity and high levels of sedentary behavior after liver and/or kidney transplants, even if they are clinically stable and have no contraindication regarding movement. This attitude persists even many years after surgery. Therefore, there is a need to increase the knowledge about the benefits of PA in the post-transplant care of children among family doctors, surgeons and the caregivers of these young patients. 

## Figures and Tables

**Table 1:** Questions and possible answers for PAQ-C and A.

Question	Response Scored 1	Response Scored 2	Response Scored 3	Response Scored 4	Response Scored 5
1. Physical activity in your spare time: Have you done any of the following activities in the past 7 days (last week)? If yes, how many times?	No	1–2	3–4	5–6	7 times or more
2. In the last 7 days, during your physical education (PE) classes, how often were you very active (playing hard, running, jumping, throwing)?	I don’t do PE	Hardly ever	Sometimes	Quite often	Always
3. In the last 7 days, what did you do most of the times at the access?	Sat down (talking, reading, doing schoolwork)	Stood around or walked around	Ran or played a little bit	Ran around and played quite a bit	Ran and played hard most of the time
4. In the last 7 days, what did you normally do at lunch (besides eating lunch)?	Sat down (talking, reading, doing schoolwork)	Stood around or walked around	Ran or played a little bit	Ran around and played quite a bit	Ran and played hard most of the time
5. In the last 7 days, on how many days right after school, did you do sports, dance, or play games in which you were very active?	None	1 time last week	2 or 3 times last week	4 times last week	5 times last week
6. In the last 7 days, on how many evenings did you do sports, dance, or play games in which you were very active?	None	1 time last week	2 or 3 times last week	4 or 5 last week	6 or 7 times last week
7. On the last weekend, how many times did you do sports, dance, or play games in which you were very active?	None	1 time	2 or 3 times	4 or 5 times	6 or more times
8. Which one of the following describes you best for the last 7 days?	All or most of my free time was spent doing things that involve little physical effort	I sometimes (1–2 times last week) did physical things in my free time (e.g., played sports, went running, swimming, bike riding, did aerobics)	I often (3–4 times last week) did physical things in my free time	I quite often (5–6 times last week) did physical things in my free time	I very often (7 or more times last week) did physical things in my free time
9. Mark how often you did physical activity (like playing sports, games, doing dance, or any other physical activity) for each day last week	None	Little bit	Medium	Often	Very often
10. Were you sick last week, or did anything prevent you from doing your normal physical activities?	Non-evaluable score	Non-evaluable score	Non-evaluable score	Non-evaluable score	Non-evaluable score

**Table 2:** Population features.

	Mean ± SD/Number
Male	21
Female	28
Age (years)	13.3 ± 3.5
Body Mass Index (kg/m^2^)	20 ± 3.6
Liver Transplantation	32
Kidney Transplantation	15
Combined Liver + Kidney Transplantation	2

**Table 3:** Answers to PAQ-C/A.

	1	2	3	4	5
Question 2 (PE classes)	33%	0%	20%	24%	22%
Question 3 (recess)	37%	37%	12%	14%	0%
Question 4 (lunch time)	53%	27%	8%	12%	0%
Question 5 (afternoon)	16%	14%	45%	10%	14%
Question 6 (evening)	45%	14%	29%	6%	6%
Question 7 (weekend)	41%	31%	14%	8%	6%
Question 8 (self-perception)	24%	27%	24%	16%	8%
Mean	36%	21%	22%	13%	8%
St. Dev.	12%	12%	12%	6%	8%

Abbreviation: PE: physical education; St. Dev.: standard deviation.

**Table 4:** Average scores recorded in study population.

Variables	n	Mean	S.D.	Min	0.25	Mdn	0.75	Max
Male	21	2.51	0.89	1.00	1.90	2.30	3.10	4.40
Female	28	2.34	0.86	1.00	1.80	2.30	2.70	4.10
<14 years old	27	2.54	0.86	1.10	1.80	2.60	3.10	4.40
≥14 years old	22	2.26	0.87	1.00	1.80	2.30	2.30	4.10
Liver Tx	32	2.54	0.95	1.00	1.80	2.55	3.25	4.40
Kidney Tx	15	2.17	0.68	1.00	1.80	2.00	2.50	3.40
Combined LTx + KTx	2	2.30	0.0	2.30	2.30	2.30	2.30	2.30
<5 years from Tx	24	2.40	0.85	1.00	1.80	2.30	3.10	4.40
≥5 years from Tx	25	2.43	0.90	1.00	1.80	2.30	2.90	4.10
Underweight	18	2.67	0.80	1.30	2.00	2.70	3.10	4.40
Normal weight	26	2.32	0.94	1.00	1.50	2.30	2.90	4.10
Overweight	5	2.02	0.43	1.50	1.80	1.90	2.30	2.60

Abbreviation: n: number; S.D.: standard deviation; Mdn: median, Tx: transplant.

## Data Availability

Not applicable.
